# Human Umbilical Cord Blood Endothelial Progenitor Cell-Derived Extracellular Vesicles Control Important Endothelial Cell Functions

**DOI:** 10.3390/ijms24129866

**Published:** 2023-06-07

**Authors:** Sawssen Ben Fraj, Sina Naserian, Bileyle Lorenzini, Sylvie Goulinet, Philippe Mauduit, Georges Uzan, Houda Haouas

**Affiliations:** 1 National Institute of Applied Sciences and Technology (INSAT), Carthage University, Tunis 1080, Tunisia; sawssen_ben_fraj@yahoo.com; 2 INSERM UMR-S-MD 1197, Hôpital Paul Brousse, 94800 Villejuif, France; sina.naserian@inserm.fr (S.N.); bileyle.lorenzini@inserm.fr (B.L.); philippe.mauduit@inserm.fr (P.M.); 3 LR18ES40, Inflammation, Environment and Signalization Pathologies, Faculty of Medicine, University of Monastir, Monastir 5000, Tunisia; 4 CellMedEx, 94100 Saint Maur Des Fossés, France

**Keywords:** extracellular vesicles (EVs), endothelial progenitor cells (EPCs), intracellular communication

## Abstract

Circulating endothelial progenitor cells (EPCs) play a pivotal role in the repair of diseases in which angiogenesis is required. Although they are a potentially valuable cell therapy tool, their clinical use remains limited due to suboptimal storage conditions and, especially, long-term immune rejection. EPC-derived extracellular vesicles (EPC-EVs) may be an alternative to EPCs given their key role in cell–cell communication and expression of the same parental markers. Here, we investigated the regenerative effects of umbilical cord blood (CB) EPC-EVs on CB-EPCs in vitro. After amplification, EPCs were cultured in a medium containing an EVs-depleted serum (EV-free medium). Then, EVs were isolated from the conditioned medium with tangential flow filtration (TFF). The regenerative effects of EVs on cells were investigated by analyzing cell migration, wound healing, and tube formation. We also analyzed their effects on endothelial cell inflammation and Nitric Oxide (NO) production. We showed that adding different doses of EPC-EVs on EPCs does not alter the basal expression of the endothelial cell markers nor change their proliferative potential and NO production level. Furthermore, we demonstrated that EPC-EVs, when used at a higher dose than the physiological dose, create a mild inflammatory condition that activates EPCs and boosts their regenerative features. Our results reveal for the first time that EPC-EVs, when used at a high dose, enhance EPC regenerative functions without altering their endothelial identity.

## 1. Introduction

Circulating endothelial progenitor cells (EPCs) are immature cells that can proliferate, migrate, and eventually differentiate into endothelial cells [1,2]. They originate from the bone marrow and are found in very low numbers in the blood of healthy individuals [3]. Several studies suggest that EPC transplantation promotes the neovascularization of ischemic tissues [4,5] and wounded vessels, underlining the important role of EPCs in vascular repair [6,7,8,9]. Another study demonstrates that they could be used for the endothelialization of a bio-artificial vessel [10]. These observations confirm their crucial role in maintaining vascular integrity [11,12]. In addition to these angiogenic properties, they have been found to suppress inflammation and provide neuroprotection [10,13].

EPCs can be isolated from the umbilical cord or peripheral blood; however, cord blood EPCs (CB EPCs) generate a greater number of colonies and can be rapidly grown in vitro compared to peripheral blood EPCs (PB EPCs) [1,14]. In addition, in vivo, adult peripheral blood EPCs form unstable blood vessels, whereas umbilical cord blood EPCs form normal-functioning blood vessels that survive for more than 4 months [15]. 

Recently, it was suggested that the therapeutic effects of EPCs may be mediated by paracrine mechanisms that possibly involve extracellular vesicles (EVs), which are an important component in intercellular communication [16]. EVs are nanoparticles delimited by a lipid bilayer membrane that are secreted by all cell types [17,18]. After the International Society for Extracellular Vesicles (ISEV) and as stated in Minimal Information for Studies of EVs (MISEV2018), based on physical criteria such as size, EVs are divided into two subtypes: “small EVs” and “medium/large EVs”, with ranges of <100 nm or <200 nm for ‘small EVs’ and >200 nm for ‘medium/large EVs’ [19]. 

Indeed, EVs produced from mesenchyme stromal cells (MSCs) and cardiac progenitor cells promote angiogenesis and restore tissue function, implying that EVs play an important role in stem or progenitor cell paracrine action [20,21]. 

EVs present numerous advantages that make them a viable alternative to cell therapy, since they can be shipped and stored for long periods. Their most important characteristic in the context of regenerative medicine is their diminished immunogenicity compared to their parental cells. This is due to the lower level or the absence of transmembrane proteins such as the major histocompatibility complex (MHC) [22,23,24]. 

This study sought to shed light on the paracrine effect of EPC-EVs on the biological functions of their producing cells (CB-EPCs). This was achieved by evaluating the impact of different doses of EVs on the regenerative functions and the expression of inflammatory markers of EPCs. The eventual aim of this study was to evaluate (1) if EPC-EVs could be used to pre-treat EPCs in order to boost their regenerative functions, and (2) if EPC-EVs could be used as a substitution for EPCs in acellular therapy in case they bear comparable capacities. 

## 2. Results

### 2.1. Characterization of Human Cord Blood Endothelial Progenitor Cells (CB-EPCs) and Endothelial Progenitor Cells Extracellular Vesicles (EPC-EVs)

**Figure 1:**
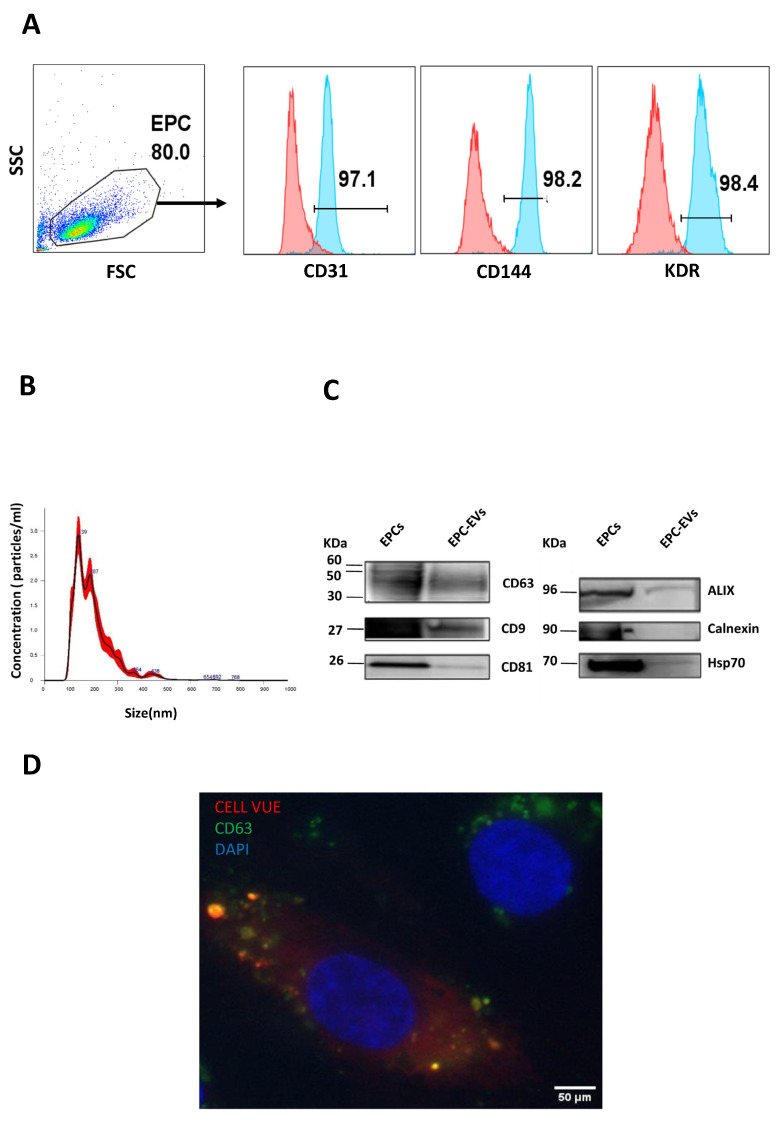
EPCs and their extracellular vesicle characterization. (**A**) Flow cytometry was used to assess the expression of endothelial surface markers by cells obtained from samples of human cord blood. Cells were positive for CD31, CD144, and KDR expression. The upper panels depict the expression of each single EPC marker, and the lower panels depict the double expression of EPC markers. (**B**) EPC-EVs were evaluated by nanoparticle tracking analysis (NTA) to determine their concentration and their size distribution. (**C**) Western blot analysis for cell lysate (EPCs) and extracellular vesicles (EPC-EVS) shows that the obtained vesicles express all the EV principal markers but not the endoplasmic reticulum marker (calnexin). The representative images are cropped sections of the total blot. Corresponding uncropped full-length blots are included in Supplementary File. (**D**) Images captured by immunofluorescence microscopy at 63X show that labeled EVs are visible in the cytoplasmic region of EPCs.

EPCs were obtained from human CB samples and characterized using flow cytometry. Colonies appeared between 7 and 20 days of culture in the EGM2 medium (endothelial cell basal medium-2 (EBM2) + EGM-2 endothelial single quotes kit containing 5% FBS. Cells were positive for EPC surface markers CD31, vascular endothelial [VE]-cadherin (CD144), and endothelial growth factor receptor-2/kinase insert domain receptor (VEGFR-2/KDR) (Figure 1A).

EVs were isolated using tangential flow filtration (TFF) from the conditioned media of EPCs cultured in an EV-free medium (EGM2 containing 5% EVs-depleted FBS). Nanoparticle tracking analysis (NTA) showed that the size of these particles ranged from 50 to 350 nm with a mean size of 179.6 nm; this is why the general term “EVs” is used throughout this manuscript (Figure 1B). 

Furthermore, Western blotting indicated the presence of the EV markers CD63, CD9, CD81, Alix, and Hsp70 in EPC and EV lysates, while the endoplasmic reticulum marker calnexin was not detected (Figure 1C and Supplementary File).

### 2.2. Endothelial Cell Internalization of EPC-EVs

To investigate the uptake of EPC-EVs by EPCs, cells were cultured for 24 h with labeled EVs. We used Cell Vue (red) and FITC conjugated antibodies (green) against CD63 to label EVs and DAPI to stain cell nuclei. Fluorescence microscopy showed the colocalization of the Cell Vue signal and CD63 dots in the perinuclear region of EPCs. This confirmed that EVs were taken up and localized in the cytoplasm (Figure 1D).

### 2.3. EPC-EVs Do Not Change the Expression of EPC Main Markers

**Figure 2:**
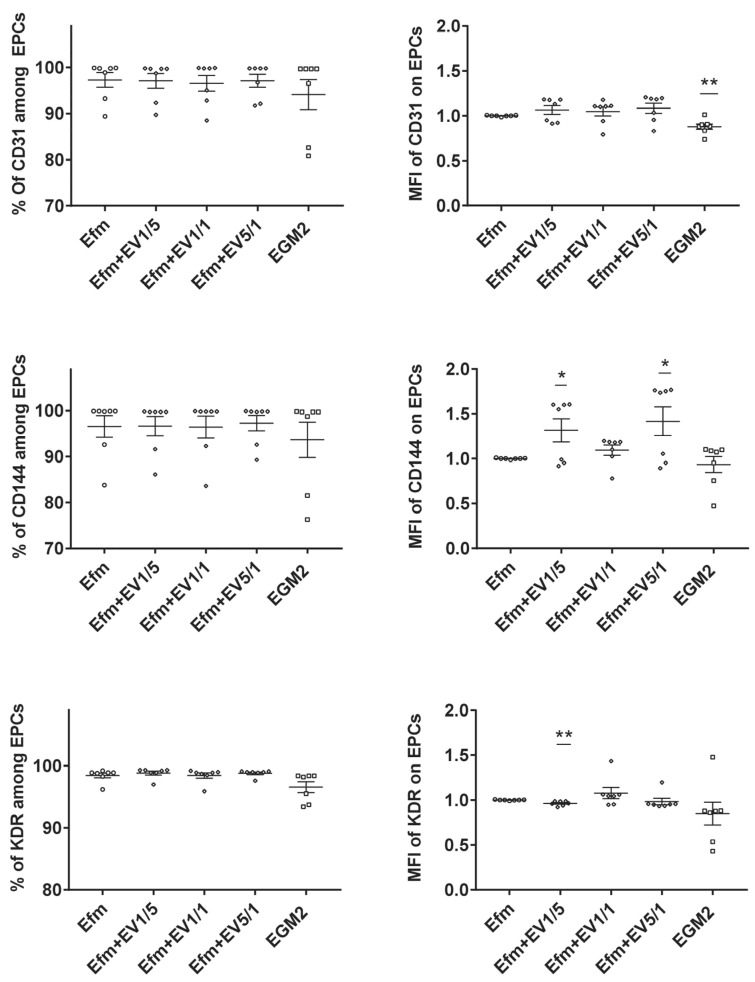
Effects of EPC-EVs on the expression of EPCs’ principal markers. EPCs were cultured for 24 h in EGM2 or EV-free medium in different (EV/cell) ratios, and their expression of endothelial markers CD31, CD144, and KDR was studied by flow cytometry. Cells cultured in an EV-free medium were considered as the control group; therefore, all other data were compared to this group. Each dot presents a measured value (*n* = 7) collected from 3 independent experiments. All values per group are expressed as a mean (±SEM). MFI values have been normalized with cells cultured in an EV-free medium (control group). An unpaired Student’s *t*-test was used to generate *p* values (* *p* < 0.05, ** *p* < 0.01). EGM2: endothelial cell basal medium-2 (EBM2) + EGM-2 endothelial single quotes kit (Lonza) + 5% FBS (Lonza). Efm: EV-free medium (endothelial cell basal medium-2 (EBM2) + EGM-2 endothelial single quotes kit (Lonza) + 5% EVs-depleted FBS).

In the next step, we assessed the effect of EPC-EVs on the expression of endothelial characteristic markers. EPCs were cultured for 24 h in the presence of several ratios of EVs (1/5, 1/1, 5/1(EVs/EPCs)). While we observed some level of variability in the expression level of CD144 and KDR, no remarkable alteration was detected in the percentage of expression of CD31, CD144, and KDR (Figure 2). These results indicate that pre-treatment with EPC-EVs does not alter the endothelial characteristics of EPCs.

### 2.4. EPC-EVs Do Not Affect EPC Proliferation and Nitric Oxide (NO) Production

**Figure 3:**
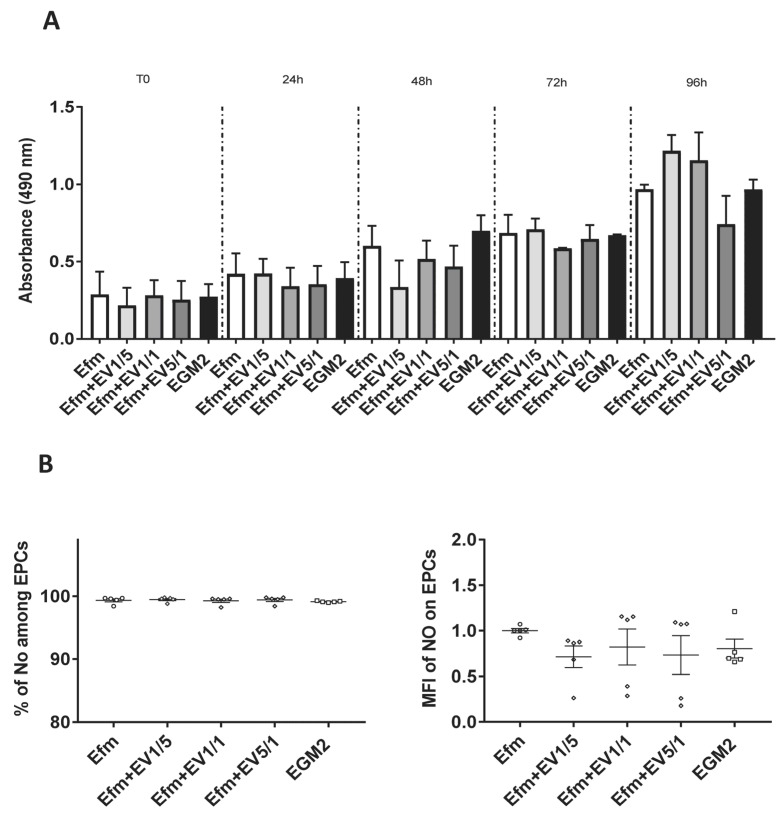
Effect of EPC-EVs on EPC proliferation and NO production. (**A**) Cells were seeded in 96-well plates with or without EVs. The absorbance was then measured after 2 h of incubation with MTS reagent and then analyzed with a plate reader (Multiskan EX, ThermoLabsystems). Cells cultured in an EV-free medium without EVs were considered as the control group; therefore, all other data were compared to this group. (**B**) To assess the impact of EPC-EVs on the NO production by EPCs, cells were cultured for 24 h in an EV-free medium with or without EVs; then, the NO production was assessed by flow cytometry and the percentage of NO-producing cells determined. Cells cultured in an EV-free medium were considered as the control group; therefore, all other data were compared to this group. Each dot presents a measured value (*n* = 5) collected from 3 independent experiments. All values per group are expressed as a mean (±SEM). MFI values have been normalized with cells cultured in an EV-free medium. An unpaired Student’s *t*-test was used to generate *p* values. (Efm: EV-free medium.).

We then assessed the impact of EPC-EVs on EPC proliferation and observed that, with different EV doses (1/5, 1/1, 5/1(EVs/EPCs), EPCs continued to proliferate and did not show a dose-dependent answer. This indicates that EVs had no toxic effects on their producing cells (Figure 3A). 

EPCs secrete an elevated amount of Nitric Oxide (NO) when subjected to stress conditions [25]. In our study, we also proved that all EPCs produced a high level of NO when stressed in deprived serum conditions (starvation in 0.2% of EVs-depleted FBS). No significant effect of EVs on NO production by EPCs was seen regardless of EV doses, meaning that EVs do not stress EPCs (Figure 3B). 

### 2.5. EPC-EVs Promote EPC Mobility and Migration

To assess the effect of EPC-EVs on EPC regenerative functions such as mobility and migration, we used wound healing and transwell migration assays.

**Figure 4:**
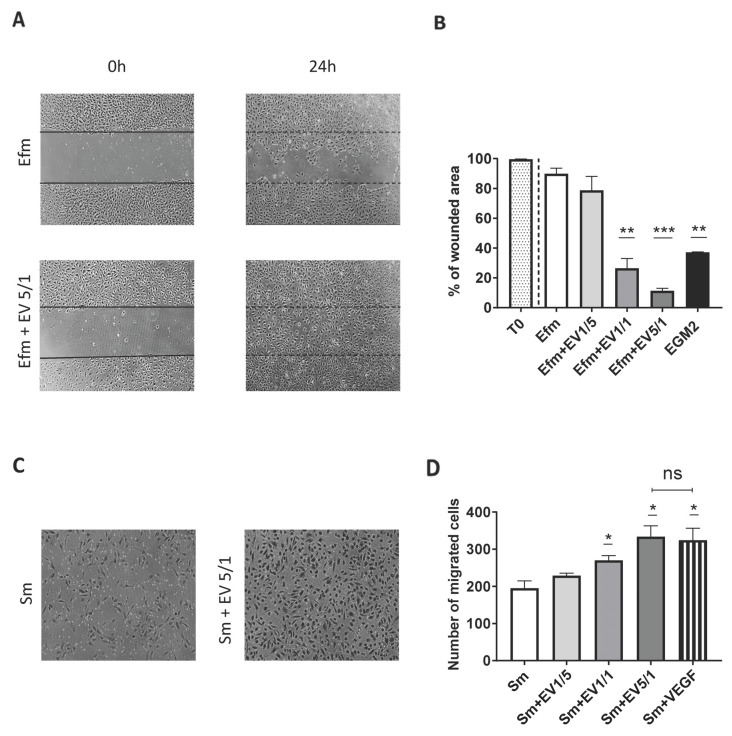
EPC-EVs promote EPC mobility and migration. EPC migration was assessed using wound healing and the transwell method. Several (EVs/EPCs) ratios were tested (1/5, 1/1, 5/1). (**A**) Pictures were taken with an inverted microscope (X4) showing the level of migration area. (**B**) Quantitative analysis of results from wound healing assay: promotion of EPCs migration is confirmed by the decrease in the scratched area when they are treated with the EVs. Cells cultured in EV-free or EGM2 medium are, respectively, considered as negative or positive control groups. (**C**) Pictures were taken with an inverted microscope (X10) showing migrated cells. (**D**) The number of migrated cells was counted using Image J software. Cells cultured in a starving medium (EBM2 (Lonza) + 0.2% EV-depleted serum) are considered the negative control group. Each dot presents a measured value (*n* = 3) collected from 3 independent experiments. All values per group are expressed as a mean (±SEM). Unpaired student *t*-test was used to generate *p* values (* *p* < 0.05, ** *p* < 0.01, *** *p* < 0.001). ns: non-significant). (0 h:0 h, 24 h:24 h, Efm: EV-free medium, Sm: starving medium.).

The wound healing assay indicated that cells treated with EVs have altered mobility function compared to control cells (Figure 4A,B). When EPCs were treated with a low dose of EVs, 1/5 (EVs/EPCs), no significant difference was observed in the wound healing function compared to the negative control (cells cultured in EV-free medium) (Figure 4B). However, higher ratios of EVs (1/1 and 5/1) significantly enhanced EPC mobility, as shown by a decrease in the wounded area in comparison with the negative control (Figure 4B). Interestingly, our data showed that cells cultured with a 5/1 EV ratio have increased wound closure capacity compared to the positive control (cells cultured in EGM2) (*p* = 0.0011) (Figure 4B).

In addition, the transwell migration assay revealed more migration capacity with EV-treated cells than untreated cells (Figure 4C). Unlike in a low ratio (1/5) condition (*p* = 0.1825), we observed a significant increase (*p* < 0.05) in the number of migrating cells with higher ratios of EVs (1/1, 5/1 (EVs/EPCs)). This confirms that only high ratios of EVs enhance EPC migration potential (Figure 4D). Since vascular endothelial growth factor (VEGF) promotes angiogenesis and endothelial cell migration, we compared its effect with that of EVs on EPC migration. Interestingly, we found no significant difference between the effect of VEGF and the 5/1 EV ratio (Figure 4D).

Taken together, these results confirm that high doses of EVs promote EPC migration and mobility, thereby suggesting an important role in vascular regeneration.

### 2.6. EPC-EVs Promote Tube Formation

**Figure 5:**
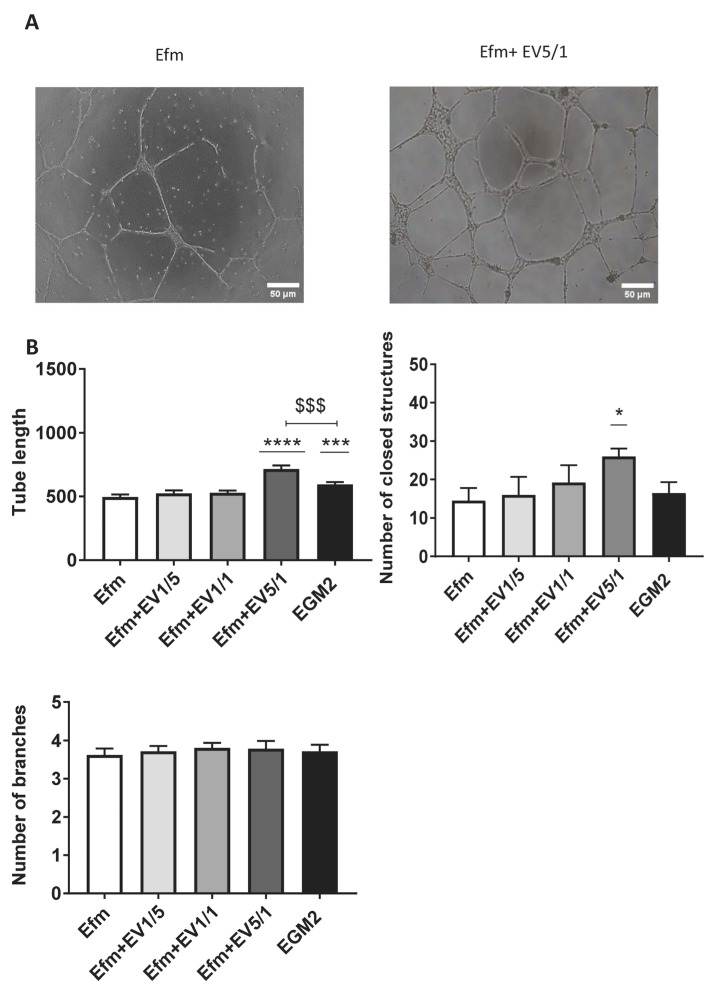
EVs enhance the network formation ability of EPCs. EPCs’ tube formation was assessed on extracellular matrix components (Geltrex). Cells were seeded with or without EVs on Geltrex. Several (EVs/EPCs) ratios were tested (1/5, 1/1, 5/1). (**A**) Images of tube formation (X10). (**B**) Quantitative analysis of tube length and the number of closed structures and branches showing that EPC-EVs enhance the network formation ability of EPCs. Results are depicted as the mean (±SEM) of 3 independent experiments. Cells cultured in EV-free or EGM2 medium are, respectively, considered as negative or positive control groups. *p* values were calculated using an unpaired Student’s *t*-test compared to the negative control group (* *p* < 0.05, *** *p* < 0.001, **** *p* < 0.0001) or to the positive control group (^$$$^ *p* < 0.001). (Efm: EV-free medium).

Endothelial cells can form tubes in the presence of extracellular matrix (ECM) components. To assess the effect of EPC-EVs on EPC tube formation, we seeded EPCs alone or with several doses of EVs on the Geltrex extracellular matrix. Several images were taken every 2 h to analyze the tube length, number of closed structures, and branching points. We found that EPCs treated with a 5/1 dose of EVs were able to form a complex tubular structure after 24 h (Figure 5A).

We observed a statistically significant increase in tube length with a 5/1 ratio (*p* < 0.0001). Untreated EPCs grown in an EGM2 medium (positive control) also showed an increase in tube length (*p* = 0.0005); however, it was statistically lower than the results seen with a 5/1 ratio (Figure 5B). 

Regarding the number of closed structures, a significant increase was also only observed with a 5/1 ratio (*p* = 0.0263). This suggests that high doses of EVs enhance the capacity of EPCs to form tubular closed networks and hence promote angiogenesis. No significant difference was detected in the number of branching points (Figure 5B).

### 2.7. Treatment of EPCs with EPC-EVs Does Not Induce an Uncontrolled Inflammatory Condition

To investigate the effect of EPC-EVs on the expression of inflammatory/activation markers, EPCs were treated with different EV doses for 24 h. We then measured the expression of the pro-inflammatory markers such as Intercellular Adhesion Molecule (ICAM), Vascular Cell Adhesion Molecule (VCAM), TIE2, and Tumor Necrosis Factor Receptor 1 (TNFR1) [26].

**Figure 6:**
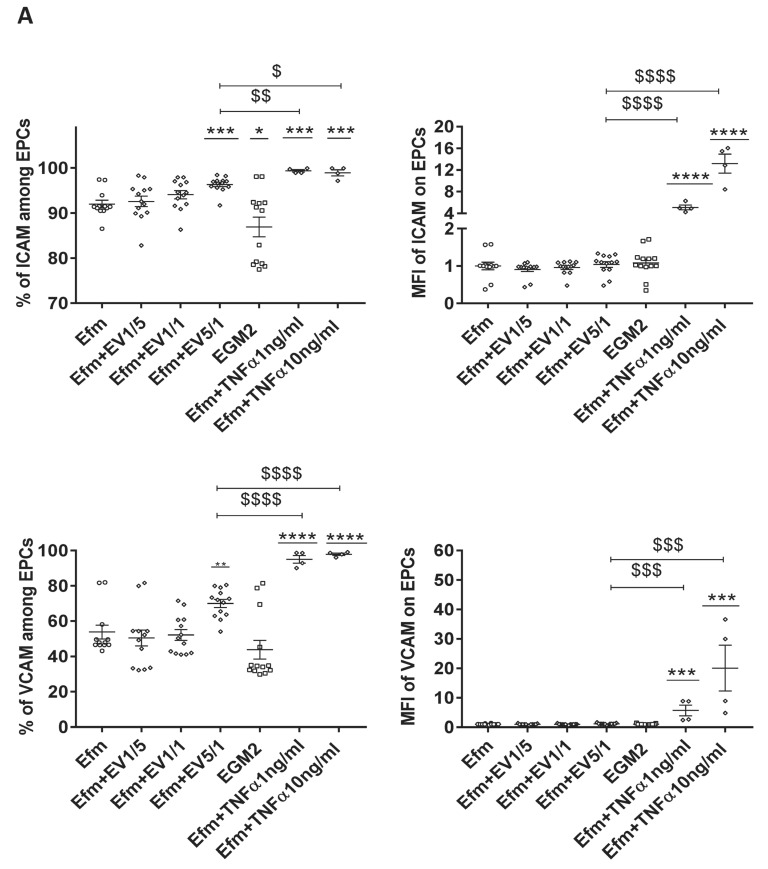

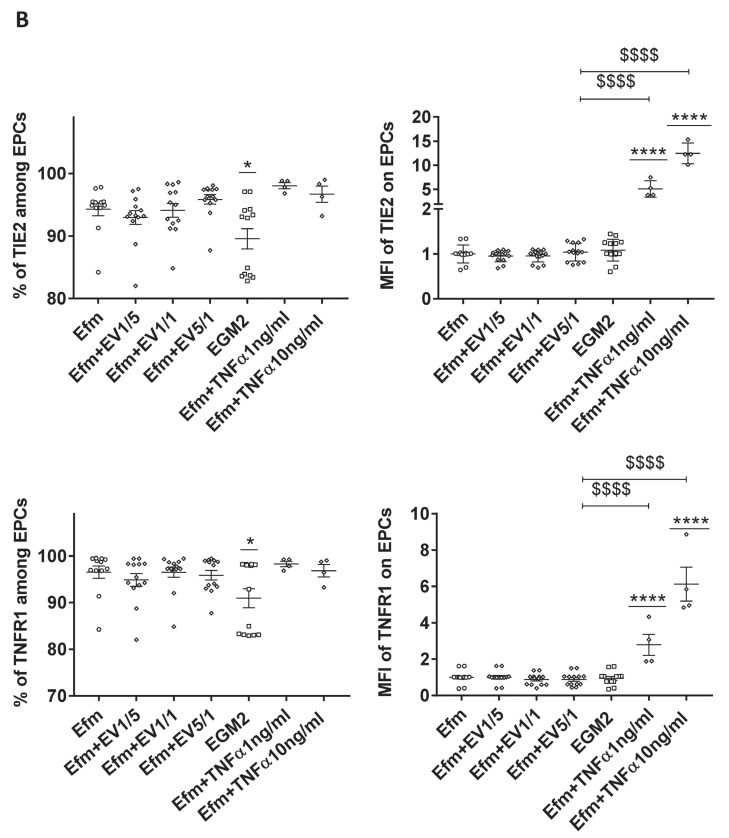
The impact of EPC-EVs on EPC inflammatory markers expression. (**A**,**B**) EPCs were cultured for 24 h in an EV-free medium with different (EVs/EPCs) ratios or with 2 doses of TNFα (1 ng/mL and 10 ng/mL), then assessed for the percentage of expression and the mean fluorescent intensity of the inflammatory surface markers ICAM, VCAM, TIE2, TNFR1. Results are depicted as mean (±SEM) of 4 independent experiments. Cells cultured in an EV-free medium without TNFα are considered the control group. MFI values have been normalized with cells cultured in an EV-free medium. An unpaired Student’s *t*-test was used to generate *p* values (* *p* < 0.05, *** *p* < 0.001, **** *p* < 0.0001) (^$^ *p* < 0.05, ^$$^ *p* < 0.01, ^$$$^ *p* < 0.001, ^$$$$^ *p* < 0.0001) (TNFα: Tumor Necrosis Factor-alpha, Efm: EV-free medium).

Culturing EPCs in an EV-free medium led to an increase in the percentage of ICAM, TIE2, and TNFR1 expression compared to the EGM2 standard medium (Figure 6A,B). This could reflect cellular stress induced by the absence of EVs. EV treatment led to a significant increase in the percentage of ICAM and VCAM expression, only with a 5/1 ratio (Figure 6A). However, this effect was not observed for TIE2 and TNFR1 expression (Figure 6B).

On the other hand, our previous study on CB-EPCs demonstrated that 1 ng/mL of the pro-inflammatory cytokine Tumor Necrosis Factor Alpha (TNFα) is the threshold between a low and an extensive inflammatory response, and that 10 ng/mL causes exacerbated inflammation [26]. Therefore, we compared the impact of different doses of EVs with that of TNFα concerning the expression of inflammatory markers. We found that both concentrations of TNFα (1 ng/mL and 10 ng/mL) led to a much greater increase in the percentage of expression of ICAM and VCAM, and the expression level of all tested markers (Figure 6A,B). Thus, EVs appear to mildly activate EPCs without causing uncontrolled inflammation.

## 3. Discussion

EPCs are involved in vascular regeneration and re-endothelialization. Accordingly, they have a crucial role in multiple ischemic diseases including myocardial infarction, stroke, peripheral arterial diseases, and disorders in which angiogenesis is required [2,27,28]. Several studies have indicated that EPCs promote angiogenesis through paracrine mechanisms [29,30,31] that may involve EVs as a mediator of intercellular communication. Our study sought to confirm this by testing the effect of EPC-EVs on their parent cells. For this purpose, we used human umbilical cord blood for the isolation of EPCs, and then to purify their EVs, since they are easily obtained and have a higher proliferative potential than EPCs obtained from adult peripheral blood [32]. 

In the first line of our experiments, we validated the EPC identity by demonstrating that they express the main endothelial cell surface markers of CD31, KDR, and CD144.

Knowing that FBS is already over-saturated by EVs, it seemed to us essential to eliminate them in our cell culture conditions in order to avoid any contamination. In this setting, all EVs were uniquely produced by our EPCs. For this purpose, we used (TFF) to isolate EVs from the serum that was used for cell culture (EVs-depleted FBS) and eventually from a conditioned medium collected from EPCs. This method led to fewer single macromolecules and aggregates than the other methods described for EV isolation, namely differential centrifugation, density gradient centrifugation, size exclusion, chromatography, ultrafiltration, immunocapture, and precipitation [19,33]. 

NTA and Western blotting showed that the purified EVs had an average size of 179.6 nm in diameter and expressed conventional EV markers such as CD9, CD63, CD81, Alix, and Hsp70. Their purity was confirmed by the absence of the endoplasmic reticulum marker, calnexin. Then, we showed that fluorescently tagged EVs were detected in the perinuclear region of EPCs 24 h after their addition to cells, indicating their internalization and intracellular cargo delivery. We also demonstrated that EVs, when used at low or high doses, do not change the expression of the main endothelial markers of CD144, CD31, and KDR.

We then investigated the effect of EPC-EVs on EPC proliferation. We showed that altering the normal physiologic ratio of EVs did not interfere with the proliferative capacity of EPCs, since they did not stop growing. Moreover, knowing that a key determinant in endothelial dysfunction is reduced NO production [25], we compared its production by EPCs in the presence or absence of EVs. Our results revealed no difference in NO production by EPCs when different EV doses were used. This reflects that the addition of EVs does not cause cellular stress and, consequently, endothelial dysfunction. 

These findings are interesting, since they suggest that the EPC-EVs could be safely used to treat EPCs without risking either any alteration in the expression of their main endothelial markers or changing their proliferation and NO production capacities. These properties are crucial when EVs are administered in vivo. 

After validating the stable phenotype and proliferative capacity of EPCs in the presence of their EVs, we aimed to investigate EPC-EVs’ impact on EPC biological functions such as mobility, migration, and network formation capacities, since they are considered important parameters in EPC pro-angiogenic effects [34,35,36,37].

Indeed, several studies showed the involvement of MSC-EVs in vascular development and their implication in angiogenesis. EVs derived from human umbilical cord MSCs improved the tube formation ability of endothelial cells in vitro, boosted their migratory properties, and promoted angiogenesis in animal cutaneous deep second-degree burn models through the Wnt4/β-catenin pathway [38]. Similarly, EVs derived from human-induced pluripotent stem cell-derived MSCs were shown to enhance the angiogenesis of human umbilical vein endothelial cells (HUVECs) [20]. Accordingly, EVs derived from adipose MSCs can also stimulate angiogenesis by delivering IL-6 to endothelial cells [39]. 

Interestingly, our results demonstrated for the first time that EPC-EVs, while used in different physiological doses, have significant functional consequences. We were able to demonstrate that high doses of EVs were able to promote EPC mobility, migration, and network formation, particularly the 5/1 ratio, whereas lower ratios were unable to promote such functions. 

Numerous studies show that EVs obtained from mesenchymal stem cells (MSCs) suppress pro-inflammatory pathways and enhance anti-inflammatory responses [40,41], primarily through diminishing the level of the inflammatory cytokines IL-1β, IL-6, and TNFα [42,43,44]. Njock et al. showed that endothelial cells suppress monocyte activation through the secretion of EVs containing anti-inflammatory microRNAs [45]. Moreover, like MSCs, EPCs are also highly sensitive to a pro-inflammatory environment [46]. Pre-treating EPCs with elevating doses of TNFα resulted in a directly related increase in the expression of endothelial activation/inflammatory markers such as ICAM, VCAM, TIE2, and TNFR1 [26]. Precisely, 1 ng/mL of TNFα could create a controlled inflammatory environment that led to EPC activation and, interestingly, to an increased immunoregulatory function. Thus, in the present paper, we wanted to compare the impact of different EV doses with increasing doses of TNFα (1 ng/mL and 10 ng/mL). Our results showed that, unlike other EV doses, the 5/1 ratio could increase the expression of ICAM and VCAM endothelial activation markers, which was always lower than that with 1 ng/mL of TNFα. Indeed, pro-inflammatory cytokine TNFα is not an optimal choice to prime EPCs, especially when targeting in vivo priming. Alternatively, our current findings make it clear that a 5/1 dose of EPC-EVs is a safe and better choice to pre-treat EPCs and prime them before their in vivo application, or even to target circulating EPCs in vivo. 

Several studies have shown that adult EPCs are dysfunctional in different cardiovascular disorders. Therefore, it is interesting to see if the administration of EPC-EVs harvested from cord blood could be an effective solution for increasing the regenerative functions of impaired adult EPCs. 

## 4. Materials and Methods 

### 4.1. Isolation and Culture of CB-EPCs 

CB samples from healthy full-term newborns were obtained from the CB Bank of St Louis Hospital (Paris, France; authorization no. AC-2022-5325). Human samples were used in compliance with the Helsinki Declaration. CB was diluted with PBS 1X containing 2 mM of EDTA before being overlaid on Pancoll (Pan biotech, Aidenbach, Germany). Mononuclear cells obtained by density gradient centrifugation were seeded into 12-well plates pre-coated with type-I rat-tail collagen (Corning, Glendale, AZ, USA), and cultured in EGM2 medium (endothelial cell basal medium-2 (EBM2) + EGM-2 endothelial single quotes kit containing 5% FBS (Lonza, Basel, Switzerland). Non-adherent cells were removed by washing with PBS 24 h later. The medium was changed every day for the first seven days and then every two days until the endothelial colony-forming cells (ECFCs: late EPCs) emerged (7–20 days). In the following tests, only cells from early passages were used (3 to 8).

### 4.2. Characterization of Human CB-Derived EPCs

Flow cytometry analysis was used to confirm the phenotype of isolated EPCs. Cultured cells were trypsinized, washed with PBS containing 3% FBS, and incubated for 20 min at 4 °C with the following antibodies: (APC)-conjugated anti-endothelial growth factor receptor-2/kinase insert domain receptor (VEGFR-2/KDR), (PE)-conjugated anti-CD144 (vascular endothelial [VE]-cadherin), and (FITC)-conjugated anti-CD31 (Miltenyi, Gladbach, Germany). Unstained cells were used as a negative control.

### 4.3. Isolation of EVs

To produce EVs, CB-EPCs obtained from several donors were cultured in an EGM2 medium. At 80% confluence, the cells were rinsed with PBS 1X and cultured for 72 h in an EV-free medium (EGM2 containing 5% EVs-depleted FBS (CellMedEX, Saint Maur Des Fossés, France). The conditioned medium was collected and centrifuged at 450 g for 5 min. EVs were then isolated using the KrosFLO KR 2i Tangential Flow Filtration system, according to the manufacturer’s recommendations (Repligen, Waltham, MA, USA). Briefly, the conditioned medium was filtered using a sterile hollow fiber filter (500 kDa cutoff). The EV containing concentrated retentate was further subjected to a polishing diafiltrated step using PBS. The purified and concentrated EVs were collected, quantified, and then stored at −20 °C until use. All these procedures were performed under supervision and according to the protocols of the EV platform of Inserm U1197.

### 4.4. Characterization of EPC-EVs

The characterization of EVs based on nanoparticle tracking analysis (NTA) and Western blotting was performed according to the validated protocols of the EV platform of Inserm U1197 [47].

NTA Nanosight NS300 (Malvern-Panalytical, Wantag, UK) was used to determine the size and concentration of EVs, as recommended by the manufacturer. The data were acquired at room temperature and evaluated using nanoparticle tracking analysis software version 3.4. The exact number of produced particles per cell was calculated based on the final EV concentration and the final number of cells. For our experiments, we used different doses of EVs according to the number of cells used in each assay. For example, the 1/1 ratio corresponds to the total EV production of one cell in 72 h, here 3832 particles/1 EPC.

Western blotting was used to analyze the characteristic markers of EVs, CD9, CD63, CD81, Alix, Hsp70, and calnexin, as recommended by the ISEV in MISEV 2018 [19]. Protein extracts of both the EV sample and cell lysates were separated by SDS-PAGE on Bolt^TM^ 4 to 12% Bis-Tris PLUS gel (Thermofisher, Waltham, MA, USA), at 100 V, for 90 min. Proteins were transferred onto a PVDF membrane using the trans-Blot turbo transfer system (Bio-Rad, Hercules, CA, USA) for 7 min. The membrane was blocked by incubation for 1 h in a blocking buffer (PBS 1X—tween 0.1%—nonfat dried milk 5%). The membrane was then incubated with primary antibodies against CD9 or CD81 (Biolegend, San Diego, CA, USA), CD63 (Invitrogen, Waltham, MA, USA), Hsp70 (RD systems, Minneapolis, MN, USA), Alix (Novusbio, Woburn, MA, USA), or calnexin (Elabscience, Houston, TX, USA) diluted to 1/1000 in (PBS 1X—tween 0.1%—non-fat dried milk 0.3%) overnight at 4 °C. After 3 × 10 min washes at room temperature with (PBS 1X—Tween 0.1%), the membrane was incubated with StarBright Blue 700 secondary antibodies (Bio-Rad, Hercules, CA, USA) diluted to 1/2500 in (PBS 1X—tween 0.1%—non-fat dried milk 0.3%). The antibody–antigen complexes were then imaged using the digital imager ChemiDoc^TM^ MP Imaging System (Bio-Rad, Hercules, CA, USA).

### 4.5. Cellular Uptake of Labeled EVs

EVs were labeled with CellVue Claret Far Red (Sigma Aldrich, Saint-Louis, CA, USA), according to the manufacturer’s instructions. To remove the excess dye, the EVs were collected by two rounds of ultracentrifugation at 100,000 g, for 1 h, at 4 °C. EVs were stained for 45 min at 4 °C with (FITC)-conjugated antibodies against CD63 (Miltenyi, Gladbach, Germany), then collected by ultracentrifugation as above and suspended in an EV-free medium. The labeled EVs were then added to cells that had been cultured for 24 h at 37 °C. After 24 h of incubation, the cells were fixed in 4% PFA and stained with DAPI, and the interaction between EPCs and EPC-EVs was visualized using a fluorescence microscope (Leica Microsystems, Wetzlar, Germany).

### 4.6. The Impact of EPC-EVs on Principle EPCs’ Surface Protein Markers Expression

CB-EPCs were seeded on 12-well plates in EGM2 medium and incubated at 37 °C, 5% CO_2_. At 80 % confluence, the cells were washed two times with PBS; then, the EV-free medium was added with different doses of EVs (1/5, 1/1, 5/1 (EVs/EPCs)). After 24 h, the cells were trypsinized, and EPC surface protein marker expression was then analyzed as described above.

### 4.7. Proliferation Test

EPCs proliferation was assessed by MTS assay. For this purpose, 5 × 10^3^ cells were seeded per well in five 96-well plates in EV-free medium supplemented with different doses of EVs (1/5, 1/1, 5/1 (EVs/EPCs)), and then incubated at 37 °C, 5% CO2. MTS (Promega, Madison, WI, USA) was diluted with EV-free medium in the dark (20 µL of MTS + 100 µL of EV-free medium for each well). After MTS was added, the plate was incubated for 2 h at 37 °C and 490 nm and 630 nm absorbance measured, as suggested by the manufacturer. The first plate (P0) was analyzed 5 h after seeding. The other plates were analyzed 24, 48, 72, and 96 h after seeding. Readings were corrected for the absorbance background in the wells without cells.

### 4.8. Nitric Oxide Production Assay

To assess NO production, CB-EPCs were cultured in EGM2 to reach 90 % confluence. After washing with PBS, cells were starved in a starvation medium (EBM2 basal medium (Lonza, Basel, Switzerland) without growth factors) containing only 0.2% EV-depleted serum for 4 h (CellMedEx, Saint Maur Des Fossés, France). Thereafter, the cells were washed with PBS and treated with different EV ratios in an EV-free medium. Untreated cells cultured in EGM2 or EV-free medium were used as controls. After 24 h, the cells were washed with PBS and incubated for 1 h with a 1 µM DAF probe (Thermofisher, Waltham, MA, USA). The cells were then trypsinized and resuspended in PBS containing 3% FBS. The labeling was assessed by flow cytometry (Fortessa, BD bioscience, Waltham, MA, USA) and data were analyzed in the FITC channel by FlowJo V10 software (Salem, OR, USA).

### 4.9. Wound Healing Assay

CB-EPCs were seeded at a density of 5 × 10^4^ cells/well in 12-well plates in EGM2 medium (endothelial cell basal medium-2 (EBM2) + EGM-2 endothelial single quotes kit containing 5% FBS (Lonza, Basel, Switzerland), and incubated at 37 °C, 5% CO_2_. At 90–100% confluence, 200 µL pipette tips were used to scratch the cell monolayer. The cells were washed two times with PBS and cultured in an EV-free medium (EGM2 containing 5% EVs-depleted FBS (CellMedEX, Saint Maur Des Fossés, France) supplemented with different ratios of EVs (1/5, 1/1, 5/1(EVs/EPCs)). Cells cultured in EGM2 and EV-free medium were used as controls. Scratch images of the controlled position were taken at time 0 (T0) and 24 h by a Nikon D5300 (Nikon corporation, Tokyo, Japan) and were analyzed using ImageJ software (National Institutes of Health, Bethesda, MD, USA).

### 4.10. Transwell Migration Assay

To test the effect of EPC-EVs on EPC migration, cells were starved overnight using an EBM2 basal medium (Lonza, Basel, Switzerland) without growth factors and containing only 0.2% EV-depleted serum (CellMedEx, Saint Maur Des Fossés, France). 5 × 10^4^ cells were then seeded into the upper chamber of Falcon cell culture inserts, pore size 8 µm (Corning, Glendale, AZ, USA). Chambers were placed in 24-well plates, and 1 mL of starvation medium containing VEGF or various ratios of EVs was added to the bottom well. After 5 h, cells that adhered to the upper side of the filter membrane were removed with a Q-tip. Migrating cells on the back side were then fixed with PFA 4% and stained with blue RAL 555 (RAL Diagnostics, Martillac, France). ImageJ software was used to count the number of migrating cells.

### 4.11. Tube Formation Assay

Geltrex (Thermofisher, Waltham, MA, USA) was used to test the influence of the EPC-EVs on tube formation by EPCs. Briefly, CB-EPCs were seeded on gel-coated wells and cultured in the presence of different ratios of EVs (1/5, 1/1, 5/1(EVs/EPCs). Cells cultured in an EV-free medium were used as a negative control. Tube formation images were taken every 2 h for 24 h and analyzed with ImageJ software. The total tube length, number of closed structures, and branching points were quantified to assess the tube-forming capacity of these cells.

### 4.12. Inflammation Markers

90% confluent CB-EPCs cultured in EGM2 were treated for 24 h with increasing ratios of EVs 1/5, 1/1, 5/1 (EVs/EPCs), or with TNFα (1 ng/mL and 10 ng/mL) for 24 h in EV-free medium. They were then stained with a mix of antibodies: Biotin-anti-ICAM (CD54), PE-anti-VCAM (CD106), PECY7-anti-TNFR1, FITC-anti-TNFR2, APC-anti-TIE2 (Miltenyi, Gladbach, Germany), and streptavidin-PE-cys5 (Thermofisher, Waltham, MA, USA). The labeling was evaluated by flow cytometry Fortessa (BD bioscience, Franklin Lakes, NJ, USA) and data were analyzed by FlowJo V10 software (FlowJo, LLC, Ashland, OR, USA).

### 4.13. Statistical Analysis

Statistical analyzes were performed with Prism (GraphPad) software. Data were expressed as means ± SEM. For cytometry, we normalized the MFI with cells cultured in an EV-free medium and used an unpaired Student’s *t*-test for *p* value generation. The *p* value < 0.05 was considered statistically significant (* *p* < 0.05, ** *p* < 0.01, *** *p* < 0.001, **** *p* < 0.0001).

## 5. Conclusions

In this work, we showed that EPC-EVs are highly involved in EPC biological functions in a dose-dependent manner. Our results demonstrated that EPC-EVs, when used at a 5/1 ratio, could increase EPC mobility, migration, and network formation capacities while keeping their endothelial identity. This work could pave the way for further in vivo studies, notably those that target impaired adult EPCs in different degenerative disorders.

## Data Availability

The datasets used and/or analyzed during the current study are available from the corresponding author on reasonable request.

## Supplementary Materials

The supporting information can be downloaded at: https://www.mdpi.com/article/10.3390/ijms24129866/s1.
