# Supplementary material for: Human Umbilical Cord Blood Endothelial Progenitor Cell-Derived Extracellular Vesicles Control Important Endothelial Cell Functions
Source: Int J Mol Sci. 2023 Jun 7;24(12):9866. doi: 10.3390/ijms24129866 (PMC10297996; doi:10.3390/ijms24129866)
Supplement: Supplementary file 1 [file ijms-24-09866-s001.zip › ijms-2409988-supplementary.pdf]

In order to characterize the extracellular vesicles (EVs), we performed a western blot using two polyacrylamide gels. We loaded an equal amount of proteins from both the EVs and cell lysates in each well. After transferring the proteins onto 2 PVDF membranes, we initially stained the membranes with Ponceau S solution to facilitate the visualization of the protein bands that were transferred onto the membrane and to evaluate the efficiency and uniformity of the protein transfer. Subsequently, images of entire membranes 1 and 2 were captured using the digital imager ChemidocTM MP Imaging System (BIO-RAD).

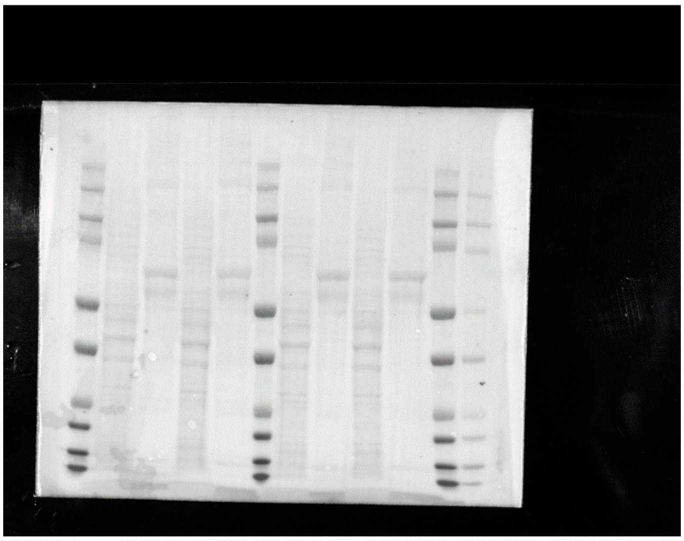

Ponceau S Stained PVDF Membrane (Gel 1)

Wells loaded from left to right:
Lane 1: Protein standard marker
Lane2: Cell Lysate
Lane 3: Extracellular Vesicle
Lane 4: Cell Lysate
Lane 5: Extracellular Vesicle
Lane 6: Protein standard marker
Lane 7: Cell Lysate
Lane 8: Extracellular Vesicle
Lane 9: Cell Lysate
Lane 10: Extracellular Vesicle
Lane 11: Protein standard marker
Lane 12: Protein standard marker

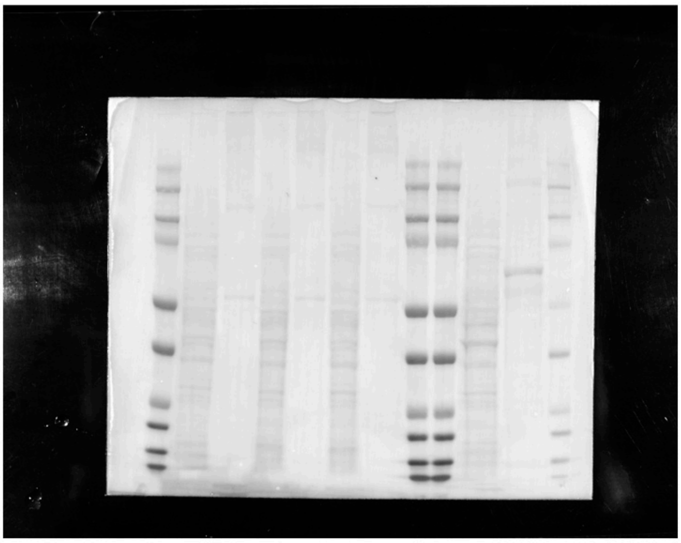

Ponceau S Stained PVDF Membrane (Gel 2)

Wells loaded from left to right:
Lane 1: Protein standard marker
Lane2: Cell Lysate
Lane 3: Extracellular Vesicle
Lane 4: Cell Lysate
Lane 5: Extracellular Vesicle
Lane 6: Cell Lysate
Lane 7: Extracellular Vesicle
Lane 8: Protein standard marker
Lane 9: Protein standard marker
Lane 10: Cell Lysate
Lane 11: Extracellular Vesicle
Lane 12: Protein standard marker

In the next step, each membrane was divided directly into multiple bands using a ruler. This approach was employed to facilitate independent incubation of each band with primary and secondary antibodies.

For the first gel, the membrane was cut into 4 bands. Each band was then, individually incubated with a primary antibody and then with a secondary antibody:

- The 1st band corresponding to lanes 1,2, and 3 was incubated with antibody against Alix

ALIX

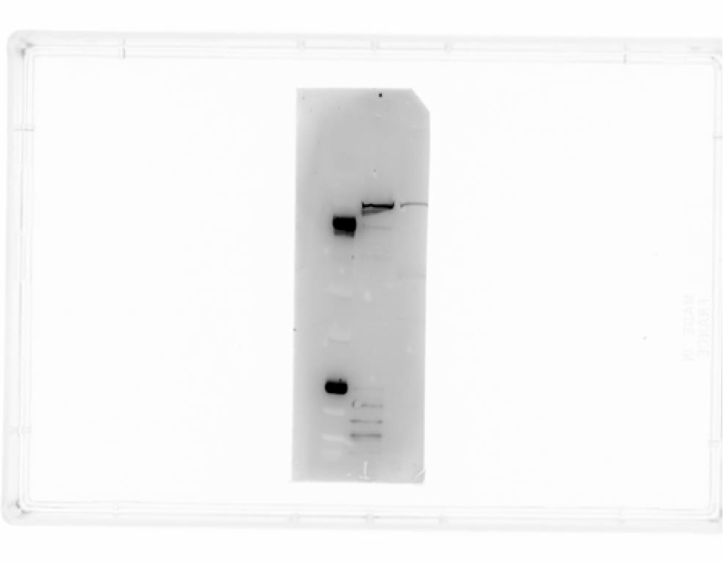

- The 2nd band corresponding to lane 4 and 5, was incubated with antibody against HSP70

HSP70

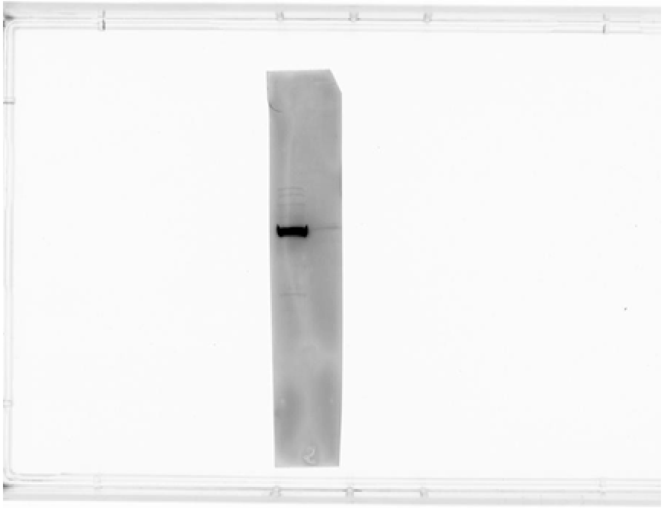

- The 3rd band corresponding to lanes 6,7, and 8 was incubated with antibody against Tubulin*

Tubulin

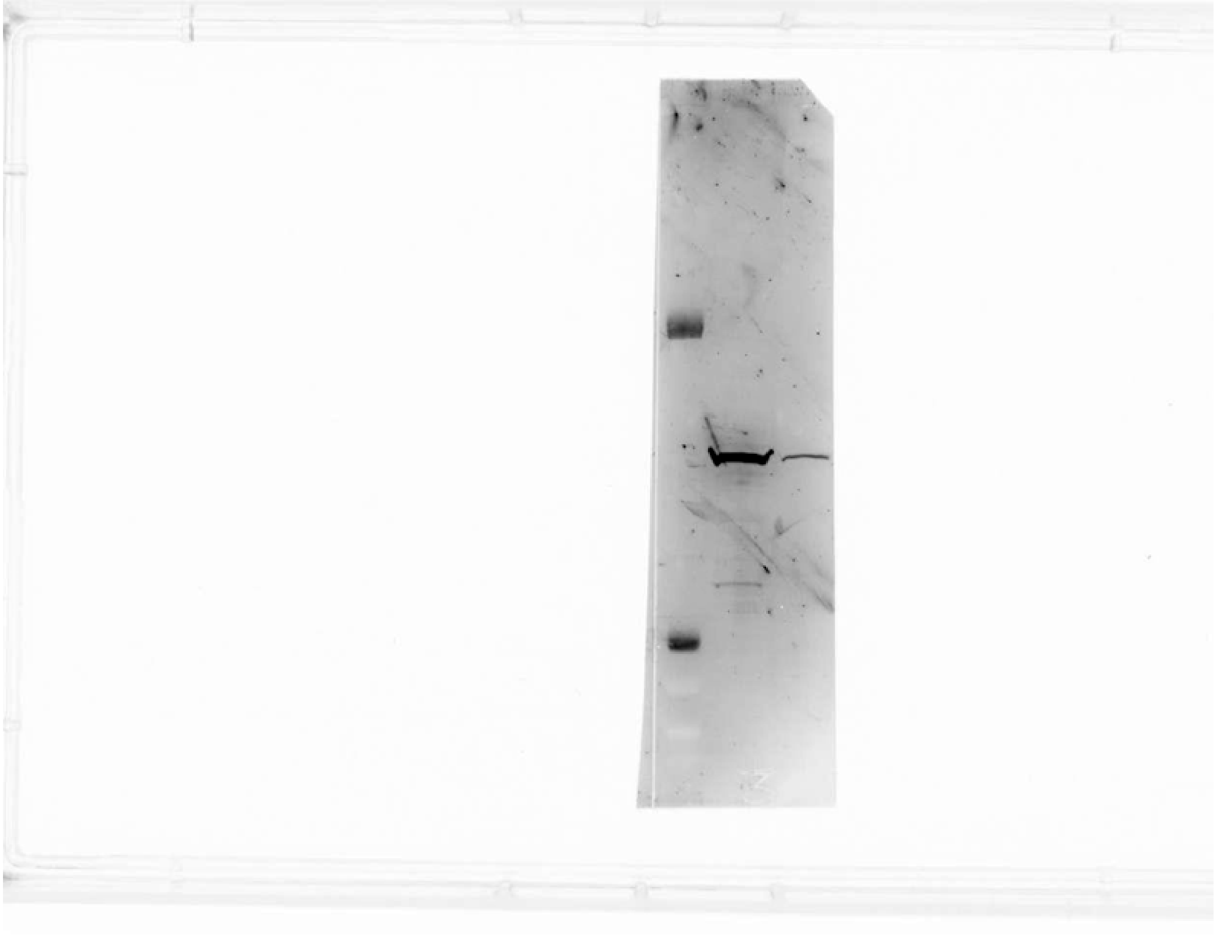

The 4th band corresponding to lanes 9,10, and 11, was incubated with antibody against Calnexin

Calnexin

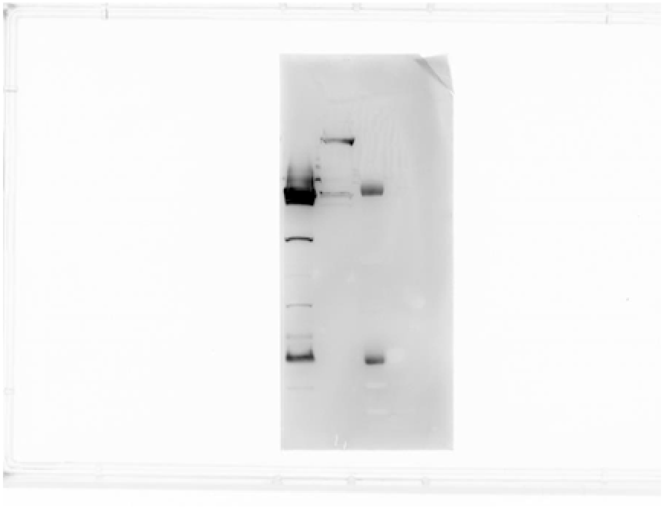

This figure represents the original image obtained, from the digital imager ChemidocTM MP Imaging System, displaying all the bands of the first gel which appeared in the supplementary figure S1.

Gel 1

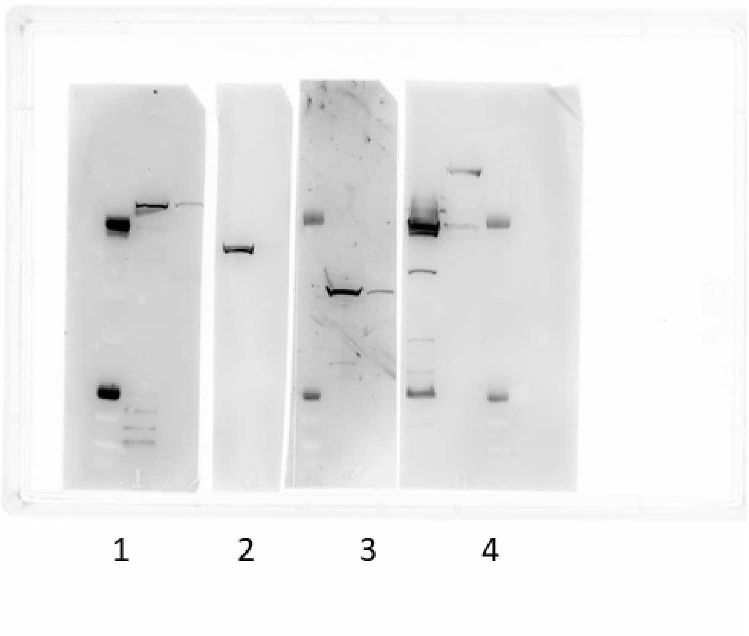

1: ALIX
2: HSP70
3: Tubuline
4: Calnexin

(*) the image coming from this band does not appear in the article.

For the second gel, the membrane was cut into 3 bands. Then each band was individually incubated with a primary antibody and then with a secondary antibody:

- The 1st band corresponding to lanes 6,7 and 8 was incubated with antibody against CD63.
- The 2nd band corresponding to lanes 4 and 5 was incubated with antibody against CD81.
- The 3rd band which corresponding to lanes 1,2, and 3 was incubated with antibody against CD9.

This figure corresponds to the original image obtained from the digital imager ChemidocTM MP Imaging System displaying all the bands of the second gel which appeared in the supplementary figure S1.

Gel 2

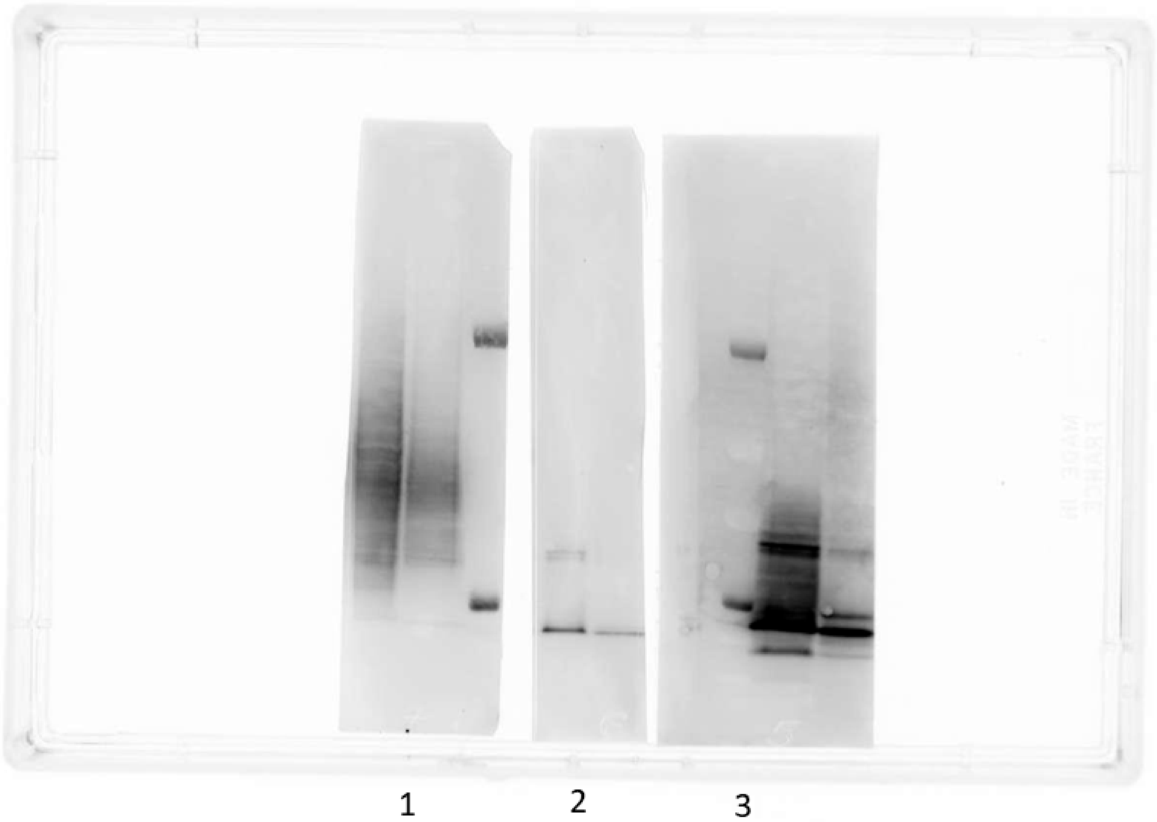

1:CD63
2:CD81
3:CD9

All images were acquired using the digital imager ChemidocTM MP Imaging System (BIO-RAD).

The proteins of interest detected on each band were used for the editing of figure 1C of the article.

In the supplementary figure S1 of the article, the original images obtained from the device were compiled without any alterations.
